# The first geospatial dataset of irrigated fields (2020–2024) in Vojvodina (Serbia)

**DOI:** 10.1038/s41597-025-04443-9

**Published:** 2025-01-18

**Authors:** Mirjana Radulović, Miljana Marković, Sanja Brdar, Ioannis Athanasiadis, Gordan Mimić

**Affiliations:** 1 BioSense Institute - the Research and Development Institute for Information Technologies in Biosystems, University of Novi Sad, Novi Sad, Serbia; 2 Wageningen University and Research Centre, Wageningen, Netherlands

**Keywords:** Environmental sciences, Hydrology

## Abstract

Irrigation is a cornerstone of global food security, enabling sustainable agricultural production and helping to ensure that food is available for people around the world, now and in the future. Mapping irrigated fields provides valuable information for sustainable water management, agricultural development, and environmental conservation efforts. However, the collection of high-quality training data, which is necessary for accurate irrigation mapping remains costly and labour-intensive. To address this, we created a georeferenced regional dataset consisting of location, crop type, and occurrence of the irrigation equipment which are essential information for mapping irrigated fields. Four main irrigated crops were considered: maize, soybean, sugar beet, and wheat. The dataset, consisting of a total of 1256 parcels, is created for Vojvodina, the main agricultural area in Serbia, spanning the period of five years (2020–2024). This study’s goal is to give accessibility to our dataset which further can be explored and used for building or fine-tuning machine learning and deep learning models for the automatic detection of irrigated fields using satellite imagery.

## Background & Summary

To meet the growing demand for food, agricultural production has to be raised to a higher level^1,2^. Irrigation is essential for maximizing agricultural productivity and ensuring food security, particularly in regions where water scarcity is a challenge. Irrigation contributes to 40% of global food production with only 20% of the total cultivated land. Still, irrigation is the largest water use sector with 87% of global water consumption which implies on large pressure on water^3,4^. Given the significant role of irrigation in food production, it is imperative to prioritize the planning of optimal water usage for irrigation. Understanding the spatial distribution of irrigated parcels represents the initial step in enhancing both agricultural and water management practices.

With a flat landscape, more than 1.6 million hectares of arable land^5^, and a long channel network of the Hydro system Danube-Tisza-Danube, Vojvodina, the main agricultural area in Serbia, has great potential for the development of irrigation. Considering channel infrastructure and water availability, it is possible to irrigate more than 50% of arable land^6^ in this region. However, despite this substantial potential, the actual extent of irrigated land in Vojvodina remains very low. In previous research, Radulović *et al*.^7^ published a Random Forest model for the classification of irrigated and non-irrigated parcels in the Vojvodina region using collected ground-truth data and Sentinel-2 images. The classification was done for the period 2020–2022 for three crop types: maize, soybean, and sugar beet. Ground-truth data were collected during the campaigns and all details will be described in this research. According to Radulović *et al*.^7^, the percentage of detected three the most irrigated crops in this region varied from 1.30% to 3.35% in the period 2020–2022. With frequent natural hazards such as drought and uneven distribution of precipitation, this agricultural region will face climate change and the need for more irrigation applications. To increase production under irrigation, but to pay attention to water availability, irrigation management should be raised to a higher level. Having that in mind, annual monitoring of irrigated areas can be a huge benefit for further irrigation planning.

The lack of comprehensive data for irrigation monitoring poses a significant challenge. Collecting data on the field is time-consuming and financially demanding which usually inhibits research as well as development methods for easier monitoring. Existing databases are usually outdated or without valuable information, such as installed irrigation capacity and their location and distribution^8^. Thus, collecting data on the field and using them to develop some quick and efficient method for automatically detecting irrigated fields has a huge benefit not only for research, but also for commercial usage, decision-makers, government, and other interested stakeholders.

To encourage research in this topic as well as to help with irrigation monitoring in the region of interest but also other neighbouring regions, we collected a dataset of irrigated and non-irrigated parcels for four crops: maize, soybean, sugar beet, and wheat in Vojvodina for the period 2020–2024. A dataset of 1256 irrigated parcel samples was created during extensive field campaigns each year. Our sample dataset will increase in volume as soon as new field campaigns take place. This data can help in developing or fine-tuning some more precise and robust machine learning or deep learning models, applicable not only for one region but for some larger areas with similar geographical characteristics. With this, we provide the dataset for other researchers or interested stakeholders who are not able to collect field data. These data were collected in the scope of the project ANTARES (https://antaresproject.eu/) to provide consistent, timely information about the spatial distribution of irrigation necessary for efficient planning and development of irrigation in Vojvodina.

## Methods

**Fig. 1:**
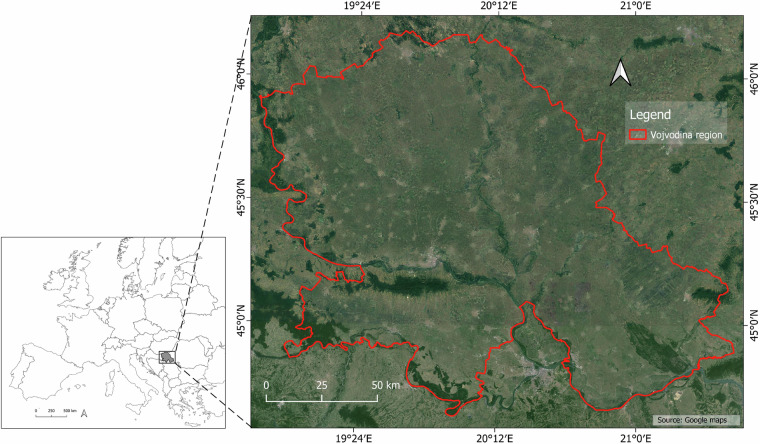
Study area of Vojvodina region.

The study area, Vojvodina, is located on the Pannonian plane, in the northern part of Serbia (44°37′–46°11′ N, 18°51′–21°33′ E) and presents the main agricultural region in this country. Located in the valleys of three rivers – Danube, Sava, and Tisza, it spans an area of 21,506 km^2^ (Fig. 1).

This region has characteristics of a moderate continental climate where winters bring cold conditions, while summers are characterized by hot and humid weather with average temperature from 21 to 23°C in July and the average annual precipitation amounts around 600 mm, with irregular annual distribution^9–11^. With predominantly flat relief and fertile soil types such as Chernozem, Vojvodina has 1.69 million hectares of arable land suitable for irrigation^5,12,13^. Among the main cultivated crops in the region, the focus of this research was on four of the most irrigated crops: maize, soybean, sugar beet, and wheat. The main sources of irrigation water in Vojvodina are surface water from the Danube and Tisza rivers, as well as groundwater.

**Fig. 2:**
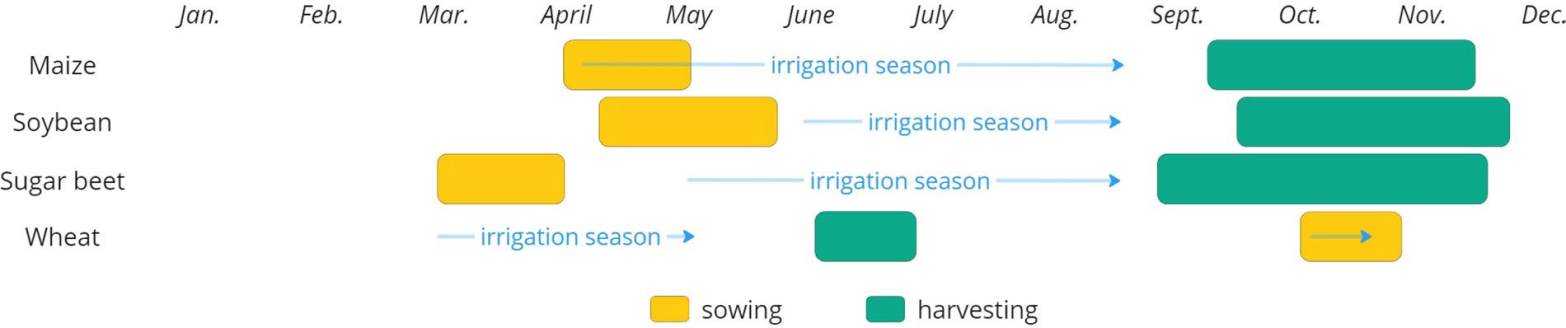
Growing calendar and irrigation season in Vojvodina for crops of interest.

Irrigation season in Vojvodina depends on the crop type (Fig. 2). Critical water requirements for maize occur during the germination and emergence phases, the vegetative stage, tasseling and silking, as well as grain filling. These phases occur from April to September. Soybean requires water in period June to September, in phases when the first flowers bloom, during pod and grain formation, and while the grains are filling. For sugar beet water is essential during intense root growth, after which water needs decrease significantly. Irrigation season for this crop last from May to September. Finally, for the wheat a critical period for water demand happens in the phase of tillering, stem elongation, flowering, and grain filling. Wheat requires water in October, immediately after sowing and than again in springtime. However, the timing and frequency of irrigation depend on various factors, including crop hybrid, sowing date, crop development, and local climate and soil conditions^14,15^.

### Ground reference data

**Fig. 3:**
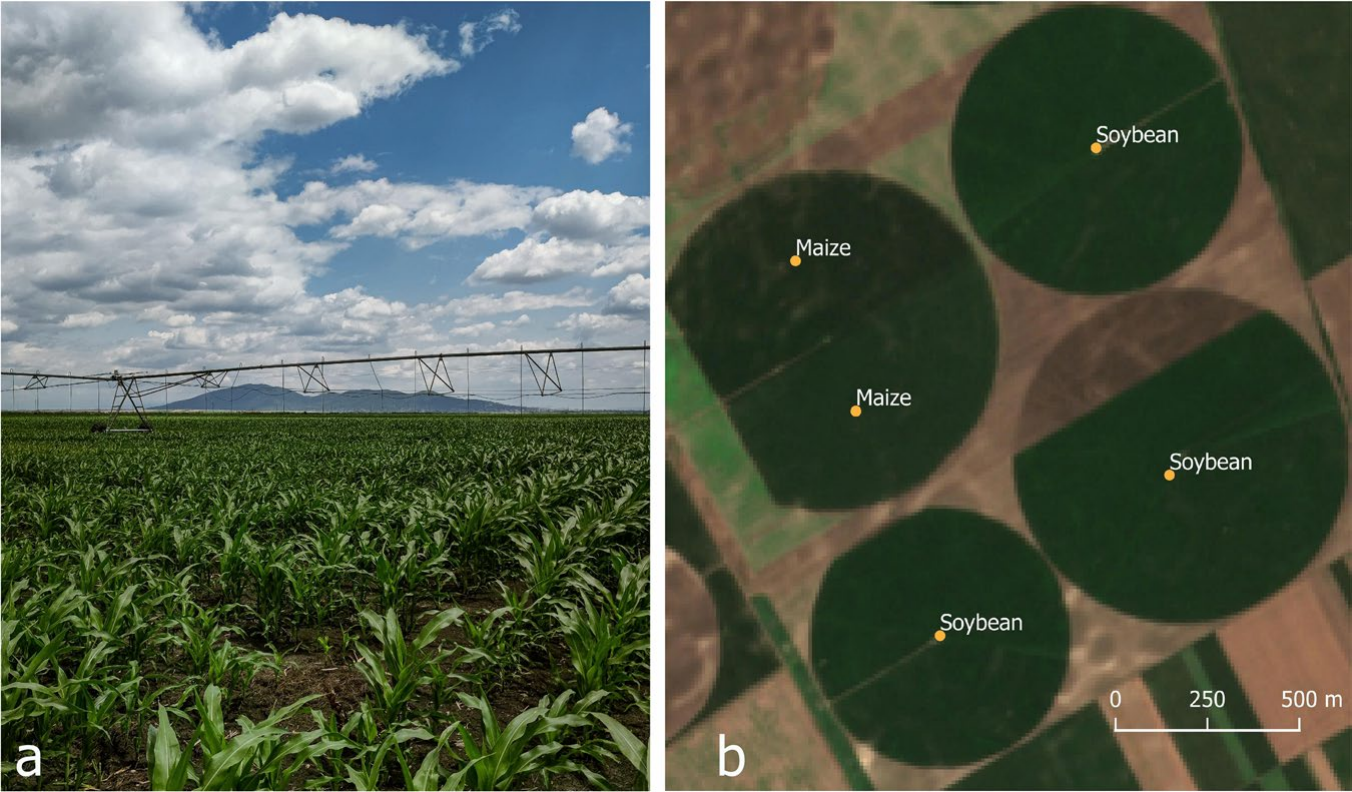
(**a**) picture of maize and irrigation system made through the smartphone application; (**b**) points generated from the georeferenced pictures.
III. **Processing of the raw data**. Lastly, the third phase of the study involved the integration of georeferenced images into QGIS software. Leveraging the rich dataset gathered during field campaigns, a polygon was drawn for each parcel, containing previously gathered information regarding crop types and irrigation status, extracted from the local database into a CSV (Comma Separated Values) file. Finally, dataset^16^ for period 2020–2024 were created containing 110 irrigated parcels in 2020, 295 in 2021, 365 in 2022, 194 in 2023, and 292 in 2024 (Fig. 4).

Five extensive field campaigns were organized during May and June in the period 2020–2024 to collect ground truth data^16^. This process included three steps:

I. **Preparation for the field campaigns**. To optimize time and gather data effectively across the expansive territory of Vojvodina, the initial phase of data gathering harnessed visual detection via Google satellite imagery. By leveraging this approach, researchers were able to pinpoint large parcels of land, with the assumption that irrigation practices were being employed on those fields. Moreover, this strategic approach to data collection ensured comprehensive coverage across all corners of the Vojvodina region, encompassing diverse relief units and soil types. This effort to capture data from various geographical and environmental contexts not only enriched the dataset but also laid the groundwork for conducting sophisticated analyses that require a comprehensive understanding of the region’s agricultural landscape.
II. **Collection during the field campaigns**. Following the initial phase of satellite-based visual detection, the subsequent stage involved on-the-ground data collection during field campaigns. These campaigns were conducted mostly in May and June when the irrigation was applied to four crops of interest. The team of researchers traversed the Vojvodina region systematically gathering georeferenced information. To facilitate this process, a specialized smartphone application, designed to capture georeferenced images, was developed in-house and employed. This innovative yet simple tool allowed researchers to capture images directly in the field, accompanied by detailed annotations regarding crop type and the presence of irrigation infrastructure (Fig. 3). During these field visits, parcels were examined to determine whether irrigation systems were installed. Parcels equipped with irrigation infrastructure (including pivot systems, linear systems, and solid set systems) were labelled as irrigated, while those lacking such infrastructure were categorized as non-irrigated.

**Fig. 4:**
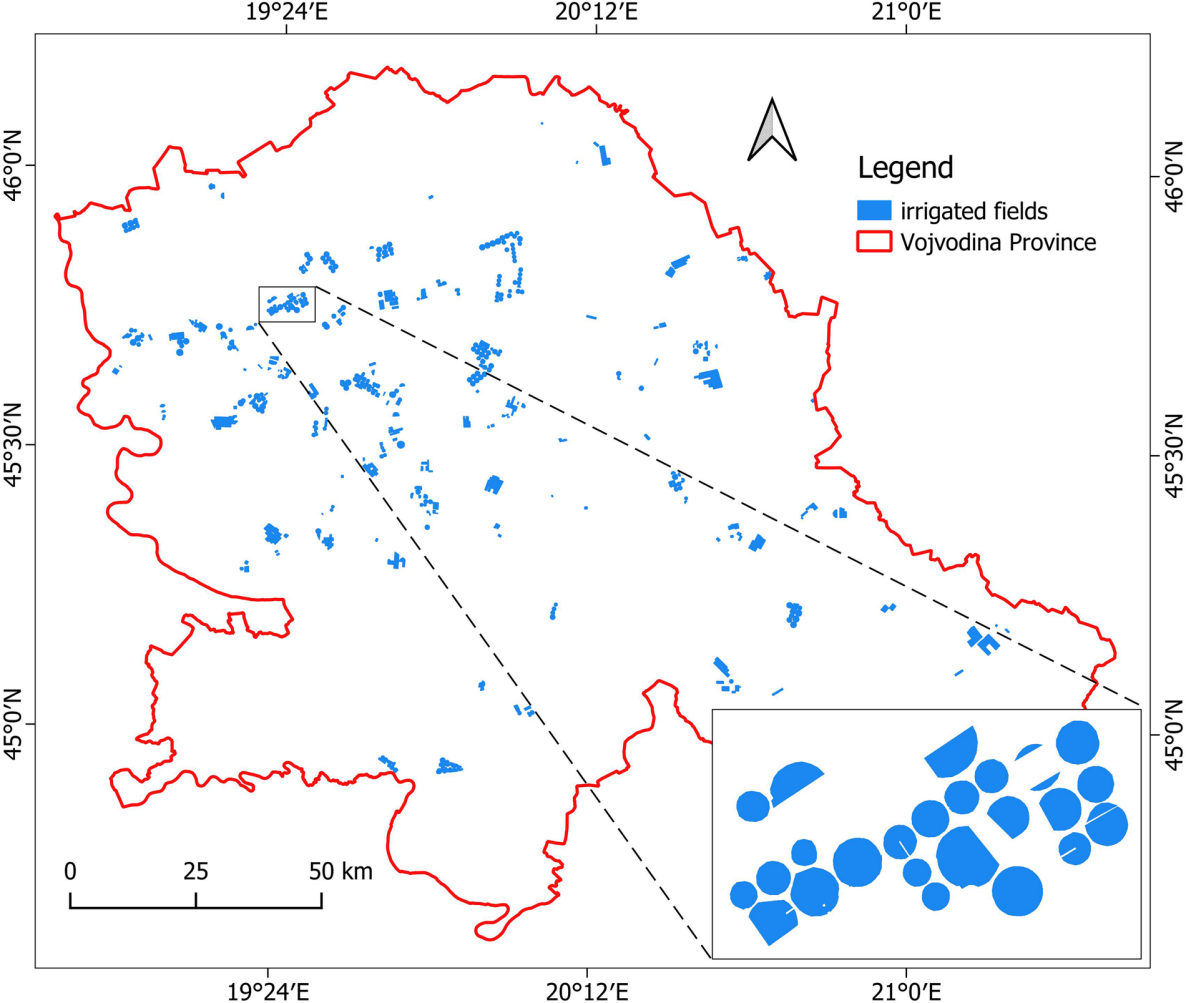
Spatial distribution of the collected ground-truth data in Vojvodina region during the period 2020–2024.

## Data Records

The annual dataset of irrigated fields for the period of five years (2020–2024) for Vojvodina region is available at the Zenodo repository^16^. It contains files: Irrigation_DataSet consisting of all irrigated samples for four crop types: maize, soybean, sugar beet, and wheat. Data are in the format of shapefile with the spatial reference system EPSG:32634. A description of the features in the attribute table is given in the “data_description.txt” file. All data could be visualized, analysed, and combined with other data. These data are useful for different analyses including the purpose of training machine learning or deep learning models to classify irrigated and non-irrigated agricultural fields using open-source satellite imagery or any other relevant data sources.

## Technical Validation

**Fig. 5:**
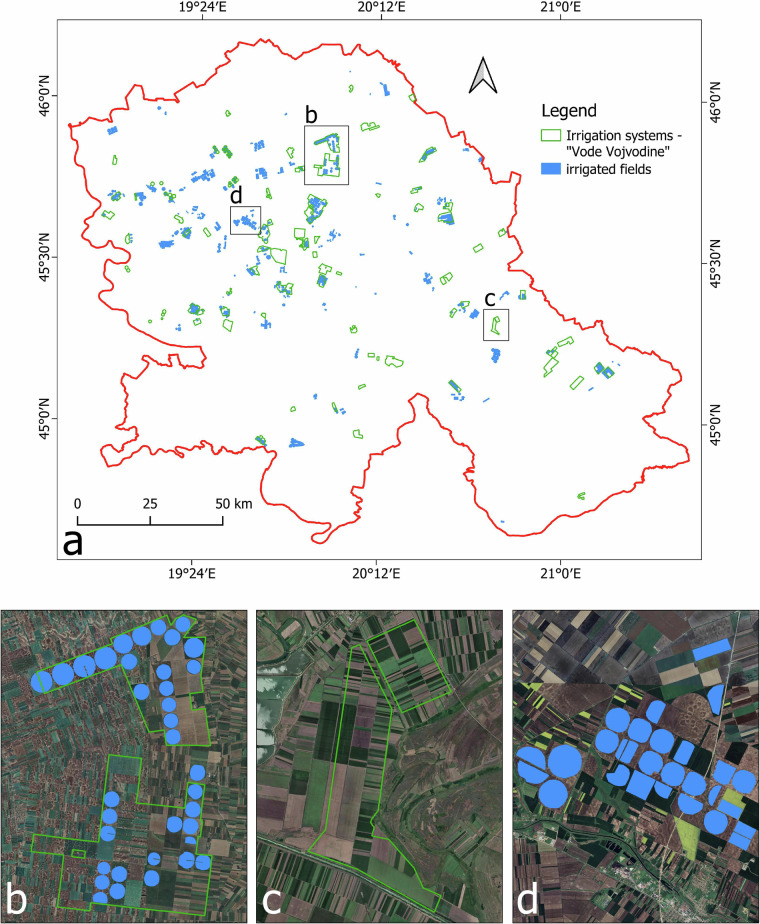
Technical validation of the collected irrigation data. (**a**) spatial distribution of irrigation systems from PWMC; (**b**) example of collected irrigation systems within PWMC polygons; (**c**) example of PWMC polygons where irrigation systems are not registered; (**d**) example of collected irrigation systems that are not in the database of PWMC.

Technical validation of the collected dataset^16^ was done using data from the Public Water Management Company “Vode Vojvodine” (further PWMC “Vode Vojvodine”). According to the last available data from 2022 year, this dataset contains 77 polygons where each polygon represents a set of several irrigated plots that farmers have registered for irrigation (Fig. 5a).

The reliability of the collected data^16^ was checked by comparing them with this dataset by overlapping and calculating how many of polygons consist at least one irrigated parcel collected during the period 2020–2024. Firstly, to avoid duplicate fields we overlapped collected data considering that if the same parcel was irrigated for more than one year, count it as one field. This dataset^16^ consists of 702 unique irrigated fields which were used for further comparison. The comparison showed that out of the total of 77 polygons from PWMC, 39 polygons consist of at least one irrigated parcel (Fig. 5b). In comparison, in 38 polygons there is no collected irrigation data (Fig. 5c). Looking at the parcel level, 244 irrigated parcels are collected within PWMC polygons. The other 458 irrigated parcels are not within any polygons (Fig. 5d) indicating that 65% of irrigated fields in the Vojvodina region are not registered in National Statistics. Thus, our dataset^16^ complements the national database on the number and location of the irrigated fields in recent years. Data from Public Water Management Company “Vode Vojvodine” that support the findings of this study through technical validation of the collected dataset^16^ were used under the licence and so are not publicly available. The data is visible on their official GIS portal (https://gis.vodevojvodine.com/smartPortal/vodeVojvodineEksterna) and are available in PDF format.

## Data Availability

To prepare all data in this study we used open source QGIS software. Other custom code was not used to generate or process the data described in the manuscript.
